# Mannose-Binding Lectin 2 Gene Polymorphism during Pandemic: COVID-19 Family

**DOI:** 10.1055/s-0042-1743258

**Published:** 2022-06-13

**Authors:** Tufan Tukek, Sacide Pehlivan, Yasemin Oyaci, Ummuhan Isoglu-Alkac

**Affiliations:** 1 Department of Internal Medicine, Faculty of Medicine, Istanbul University, Istanbul, Turkey; 2 Department of Medical Biology, Faculty of Medicine, Istanbul University, Istanbul, Turkey; 3 Department of Physiology, Faculty of Medicine, Istanbul University, Istanbul, Turkey

**Keywords:** COVID-19, mannose-binding lectin 2, polymorphism

## Abstract

Mannose-binding lectin 2 (MBL2) is a serine protease which is believed to be an important factor in the inherited immune system. In this article, we present a coronavirus disease 2019 (COVID-19) family of five patients: a 56-year-old father, a 51-year-old mother, two sons aged 23 and 21 years, and a 15-year-old daughter. According to the results of *MBL2* *rs1800450* variant analysis performed, the father had homozygous mutant, the mother had homozygous normal, and the three children had heterozygous mutant genotype. When we compared the clinical parameters and genotypes, *MBL2* gene polymorphism plays a very important role in COVID-19 susceptibility and severe disease. The family, which makes up our study, is the proof of this situation, and it contains important implications for host factors and COVID-19.

## Introduction

Coronavirus disease 2019 (COVID-19) is a pandemic that has infected many people since the first case was announced and has been among the major infectious events of the century. Individuals older than 65 years and patients with comorbid burden have a mortal course. 1 2 3

Specifically, detecting host-based factors that predispose to infection constitutes a very important research area. In this context, one of the different host factors can be mentioned from the literature: Mannose-binding lectin 2 (MBL2) is a serine protease belonging to the collectin family and is believed to be an important factor in the inherited immune system. The MBL2 protein binds to the surface of a wide range of microorganisms by its ability to recognize or function directly as an opsonin or through activation of the complement system, therefore increasing the phagocytosis of microorganisms by macrophages and neutrophils. 4 There are several known polymorphisms in the *MBL2* gene (10q21.1), located on the long arm of chromosome 10, in both the promoter and exon regions, resulting in multiple haplotypes. These genetic polymorphisms are associated with different levels of MBL expression and activity. 4 5 Various studies on the association of *MBL2* genetic polymorphism and/or MBL plasma levels with severe infections, sepsis, and septic shock have shown an increased risk of developing sepsis in patients with MBL deficiency and a negative outcome. 5 6

Medetalibeyoglu et al investigated the clinical effect of the *MBL2* ( *rs1800450* ) gene variant in a total of 284 COVID-19 cases. 7 The BB genotype of the *MBL2* gene was found to be more common among COVID-19 cases compared with healthy controls. The risk of severe disease (odds ratio [OR] = 5.3, *p* < 0.001) and intensive care unit (ICU) requirement (OR = 19.6, *p* < 0.001) were revealed to be higher for BB genotype. On the other hand, no significant difference was found between genotype variants in terms of 28-day mortality or secondary bacterial infection development.

## Methods and Results

In this article, the *MBL2* ( *rs1800450* ) genetic polymorphism was analyzed using the same method, and we present a COVID-19 family of five patients that was proven in parallel with main study. 8 A written consent was obtained from all patients for both sampling and publishing.

The COVID-19 family consisted a total of five members: a 56-year-old father, a 51-year-old mother, two sons aged 23 and 21 years, and a 15-year-old daughter. The father does not have any comorbidities. He was taken to home for isolation on April 2, 2020, after the polymerase chain reaction (PCR) sample was taken in the emergency department, where he presented with symptoms related to upper respiratory tract. Among the PCR samples taken from all patients, only he was positive. On April 7, 2020, all samples were taken again and 23-year-old son was positive. The samples of all family members, together with the father, who admitted to the emergency department on April 9, 2020, with dyspnea and refractory fever, were repeated. The father was hospitalized in the ICU to be followed up with a noninvasive mechanical ventilator in the picture of multi-inflammatory syndrome-adult (MIS-A) with respiratory failure findings. Despite bilateral diffuse lung involvement, the mother without any known comorbidity was taken to the ward due to her vital parameters and clinical stable status. The PCR result taken in the emergency department was positive. The 23-year-old son was hospitalized with bilateral diffuse lung infiltration in the MIS-A picture in ICU. Repeated PCR test is also positive. Of the other two children, the 21-year-old son also showed bilateral lung infiltration, but was only isolated in hospital. The PCR test result was positive. On the other hand, the first PCR test taken in the emergency department of the 15-year-old daughter was negative, but it was decided to be followed up in the hospital due to high risk of contact, and the test repeated 2 days later was found to be positive. The father received anticytokine and anticoagulant treatment during the ICU follow-up; he was discharged after 10 days. The mother and the 23-year-old son were discharged after 7 days with only anticoagulant and supportive treatment, while the 21-year-old son and the 15-year-old daughter were followed up with home isolation after 4 days of anticoagulant and supportive treatments in the hospital. Clinical findings and initial laboratory results are shown in Table 1 .

**Table 1:** Initial clinical and laboratory findings of the family

	Father	Mother	23-year-old son	21-year-old son	15-year-old daughter
Date of PCR positivity	April 2, 2020	April 9, 2020	April 7, 2020	April 9, 2020	April 11, 2020
Date of hospitalization	April 9, 2020	April 9, 2020	April 9, 2020	April 9, 2020	April 9, 2020
Fever	39	38.3	37.8	37.5	37.8
ABP	138/85	125/80	130/80	120/70	120/80
HR	132	110	130	94	94
RR	26	14	16	14	16
OS	84	96	94	98	98
WBC	6,810	6,610	4,810	5,600	6,600
Hemoglobin	13.1	12.3	16.4	15.6	12.4
Neutrophil	5,070	3,400	2,870	3,550	3,600
Lymphocyte	1,290	2,800	1,560	2,000	3,000
Platelet	221,000	416,000	139,000	318,000	214,000
Urea	12	23	22	16	18
Creatinine	0.79	0.68	0.97	0.98	0.67
LDH	444	249	327	146	166
CRP	10.04	0.77	23.94	4.6	2.4
Ferritin	863	35.53	317.8	98	66
AST	42	32	43	22	20
ALT	52	22	35	24	24
D-dimer	1.2	0.13	0.08	0.01	0.01
CT findings	Bilaterally involvement	Bilaterally involvement	Bilaterally involvement	Bilaterally involvement	Bilaterally involvement
Need for ICU	Yes	No	Yes	No	No
Follow-up duration	10	7	7	4	4

Abbreviations: ABP, arterial blood pressure; ALT, alanine aminotransferase; AST, aspartate aminotransferase; CRP, C-reactive protein; CT, computed tomography; HR, heart rate; ICU, intensive care unit; LDH, lactate dehydrogenase; OS, oxygen saturation; PCR, polymerase chain reaction; RR, respiratory rate; WBC, white blood cell.

**Fig. 1:**
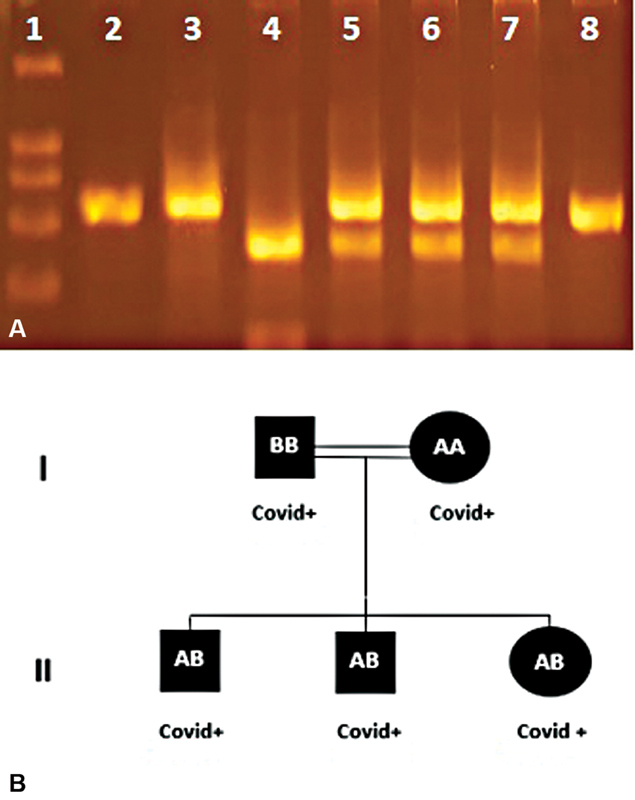
( **A** ) PCR-RFLP products of MBL2 ( *rs1800450* ) polymorphism on 2% agarose gel. ( **B** ) Family pedigree. 1. Marker, 2/8. Nondigest PCR product, 3. Father/BB, 4. Mother/AA, 5. Son/AB, 6. Son/AB, 7. Daughter/AB (BB: homozygous mutant—gene expression negative, AB: heterozygous mutant—gene expression decreased—1/10, AA: homozygous normal—gene expression positive).

According to the results of *MBL2* ( *rs1800450* ) variant analysis performed retrospectively, father had BB (homozygous mutant), mother AA (homozygous normal) and the three children had AB (heterozygous mutant) genotypes ( Fig. 1 ).

## Discussion

To the best of our knowledge, the COVID-19 family we have reported constitutes the first report in which *MBL2* gene variants are shown. As a summary of studies which investigate the effect of the *MBL2* genotype on gene expression, it was reported that MBL expression in the AB genotype decreased by 1:10, while there was no expression in the BB genotype. 7 9 In our previous study, it was revealed that patients with BB genotype have a more severe clinical picture.

Clinical findings are supported by *MBL2* gene polymorphisms in other studies. In a meta-analysis on severe acute respiratory syndrome coronavirus (SARS-CoV)-1, Middle East respiratory syndrome–related coronavirus, and SARS-CoV-2, 22 out of 32 articles between January 2003 and June 2020 were found to be eligible for review. 10 As a result of the analysis, it has been determined that *MBL2* gene variants are effective in at least two studies. In another study, Zhang et al examined the frequencies of one mutation in codon 54 and three promoter polymorphisms in nt 550, 221, and four in 352 patients with SARS and 392 healthy controls by using PCR direct sequencing. 11 12 In this study, codon 54 variant ( *rs1800450* ) ownership was associated with decreased MBL2 expression and SARS-CoV susceptibility. In another study, *MBL2* gene polymorphisms and MBL serum levels were examined. 12 The distribution of *MBL2* gene polymorphisms was significantly different between SARS patients and the control group, the frequency of haplotypes associated with low or missing MBL serum levels was higher in SARS patients than in the control group. Serum MBL levels were also significantly lower in SARS patients than in the control group.

In the family who was the subject of our study, it is an expected finding that the father has a BB genotype and has the most severe clinical picture. It is thought that the most severe clinical picture after the father, unlike his other siblings, is seen in the 23-year-old son with multiple reasons: he is the first person to become positive after the father and was probably disadvantaged in terms of viral exposure and viral load compared with the other siblings. Because of the gender difference and more importantly, consanguineous marriage between parents, the effects of other possible recessive genes may also have created this difference. The fact that the 15-year-old daughter had the mildest clinical picture, and the disease is thought to be related to the latest PCR positivity and less viral load exposure with isolations. We would like to highlight again that the mother is normal and has a mild life.

## Conclusion

As a result, *MBL2* gene polymorphism plays a very important role in terms of COVID-19 susceptibility and severe disease. The family, which makes up our case report, is the proof of this situation, and it contains important implications for host factors and COVID-19.
